# Longitudinal Changes in Stress and Isolation Among Multi‐Ethnic Breast Cancer Survivors Throughout COVID‐19

**DOI:** 10.1002/smi.70063

**Published:** 2025-06-18

**Authors:** Fangyuan Zhao, Jincong Q. Freeman, Nora Jaskowiak, Gini F. Fleming, Rita Nanda, Diane S. Lauderdale, Olufunmilayo I. Olopade, Dezheng Huo

**Affiliations:** ^1^ Department of Public Health Sciences University of Chicago Chicago Illinois USA; ^2^ Department of Surgery University of Chicago Chicago Illinois USA; ^3^ Department of Medicine, Section of Hematology and Oncology University of Chicago Chicago Illinois USA

**Keywords:** breast cancer survivors, longitudinal survey, mental health, social isolation, stress

## Abstract

As COVID‐19 transitions to a more manageable phase, it remains unclear whether its impact on mental health has similarly eased among cancer survivors. This longitudinal study tracked how the levels of stress and isolation experienced by breast cancer survivors (BCS) of different racial/ethnic groups have changed as the pandemic evolved. BCS enroled in the Chicago Multiethnic Epidemiologic Breast Cancer Cohort were surveyed between July and September of 2020, 2021, and 2022. An 11‐item isolation/stress score was repeatedly measured in each survey. Mixed‐effects linear regression models were used to analyse changes in the isolation/stress scores over time across different racial/ethnic groups and to identify the socioeconomic factors associated with the racial disparities observed. In total, 1899 BCS responded (response rate: 62.8%), of whom 69% were White and 24% Black. The median time from diagnosis to first survey was 5.1 years (IQR: 2.3–9.2). The isolation/stress score decreased continuously for White BCS (*P*‐trend < 0.001), but only began declining for Black BCS in the last wave of survey. Black BCS had significantly higher isolation/stress scores in 2021 and 2022 compared to Whites (both *p* < 0.01). The racial differences became insignificant after adjusting for certain socioeconomic factors. Notably, BCS who were single, on Medicaid, without a high school degree, or with annual household income less than $35,000 had significantly higher isolation/stress scores (all *p* < 0.05). The findings remained consistent in sensitivity analysis using inverse probability weighting to account for non‐response. Our findings suggested that the levels of stress and isolation of BCS did not improve equally across different racial/ethnic groups as the pandemic subsided. This may be associated with disparities in socioeconomic factors like insurance coverage, education level, income level and family composition. Understanding these barriers and challenges is crucial for developing targeted interventions and support systems for vulnerable populations as we recover from the pandemic and prepare for future health challenges.

## 1 Introduction

With COVID‐19 cases and related deaths declining to manageable levels, the pandemic has transitioned from a global health emergency to an established and ongoing public health concern (World Health Organization 2023). Nevertheless, an important question remains: has the impact of the pandemic on mental health similarly eased? In response to this question, this study focused on the longitudinal change in the mental health of cancer survivors, a population that may continue to face elevated distress even as the pandemic subsided.

At the pandemic's onset, influenced by psychological and socioeconomic stressors such as fears of illness, extended lockdowns, and economic insecurity, many studies on mental health reported a profound increase in feelings of isolation, stress, and depressive symptoms (Bower et al. 2023; Matsubayashi et al. 2022; Zamanzadeh, Cavoli, et al. 2024). However, few studies have examined the longitudinal changes in mental health as the pandemic subsided, and those that did reported inconsistent findings on whether it has recovered (Kim et al. 2023; Manchia et al. 2022; Patler and Langer 2024; Taxiarchi et al. 2023; van der Velden et al. 2023).

In this study, we are interested in examining the mental well‐being of cancer patients and survivors, who may be particularly vulnerable. Compared to the general population, cancer patients and survivors were at significantly higher risks of COVID‐19 infection, hospitalisation, and death; while delayed treatment and limited access to follow‐up care acted as additional stressors, further compromising their quality of life and long‐term survival (Muls et al. 2022; Qian et al. 2025; Wang et al. 2020). Research on the mental well‐being of cancer survivors has predominantly been cross‐sectional (Almeida et al. 2024; Muls et al. 2022; Zhang et al. 2022), with only a handful of longitudinal studies conducted at the onset of the pandemic (i.e., 20.0–2021) and limited in sample size (Bastien et al. 2023; Bethea et al. 2022; De Jaeghere et al. 2022; Han et al. 2021). Thus, the longitudinal changes in the mental well‐being of cancer survivors in the later stages of the pandemic remained unexplored.

Moreover, cancer survivors from racial/ethnic minority groups may be disproportionately affected. Racial/ethnic minorities could face additional pandemic‐related stressors, including higher exposure to infection through frontline work, greater income loss and food insecurity (Goldmann et al. 2021; Parenteau et al. 2023). Most studies also reported that racial/ethnic minorities suffered from worse mental well‐being compared to their White counterparts (Bui et al. 2021; Thomeer et al. 2023); while findings from studies specifically focused on cancer survivors have been mixed (Hamlish and Papautsky, 2022; Qian et al. 2025). In a longitudinal study of the general population, racial/ethnic minority groups experienced more persistent distress over time and showed less recovery as the pandemic subsided (Patler and Langer 2024), though it is unclear whether similar trajectories are observed among cancer survivors.

In light of these knowledge gaps, we conducted a three‐wave longitudinal survey study in 2020, 2021, and 2022 among a large multi‐ethnic cohort of breast cancer survivors (BCS). We repeatedly measured their levels of stress and isolation as the pandemic evolved over time, and assessed the presence of racial disparities. We further examined the factors associated with these disparities and discussed potential implications for public health strategies. To our knowledge, this study is the first to track the longitudinal changes in stress levels among a large, diverse cohort of cancer survivors from the onset of the pandemic through its later phases.

## 2 Methods

### 2.1 Survey Design

This three‐wave longitudinal survey study was conducted within the Chicago Multiethnic Epidemiologic Breast Cancer Cohort (ChiMEC). Between July and September of 2020, 2021 and 2022, three waves of surveys were sent out to 3023 eligible ChiMEC participants via RedCap and follow‐up phone calls, acheiving an overall response rate of 62.8%. All participants provided their written informed consent. The study was reported according to the STROBE guidelines.

### 2.2 Measures

The three surveys repeatedly measured isolation and stress using the same isolation/stress score calculated from 11 items (Zhao et al. 2021). These items were adopted from established item banks (Cella et al. 2010; Gerhold 2020) and measured on a five‐point Likert scale ranging from 0 to 4. The items showed good internal consistency in all three waves of surveys (Cronbach *α* = 0.85, 0.87, 0.89, respectively). The total score can be further decomposed into two subscales: the social isolation score (4 items; range: 0–16; Cronbach *α* = 0.70, 0.74, 0.75, respectively) and the stress score (7 items; range: 0–28; Cronbach *α* = 0.81, 0.79, 0.79, respectively).

Patient‐reported race and ethnicity were recorded following the Centers for Disease Control and Prevention Race and Ethnicity codes. Demographic and clinical characteristics (i.e., date of birth, date of diagnosis, comorbidities, tumour subtype and stage, insurance type) were extracted from electronic medical records and hospital cancer registry. Individual‐level socioeconomic status (SES) factors including number of people living in the same household, marital status, annual household income and education level were collected through surveys. To collect community‐level SES of participants, their residential addresses were geocoded to identify their census block groups and were then linked to the American Community Survey (20.6–2020) (U.S. Census Bureau, 2021) and the Area Deprivation Index (ADI, 2020) (University of Wisconsin School of Medicine and Public Health 2020).

### 2.3 Statistical Analysis

Standard descriptive statistics were used to compare the distribution of patient characteristics across racial/ethnic groups, using *t*‐tests for normally‐distributed continuous variables, Wilcoxon rank‐sum tests for skewed continuous variables and ordinal variables, and χ^2^ tests for categorical variables. All tests of statistical significance were 2‐sided, with *p* < 0.05 considered statistically significant. Mixed‐effects linear regression models were used with the isolation/stress score as the outcome, incorporating random intercepts to account for within‐person correlation due to repeated measurements. To assess whether the changes in isolation/stress scores over time differed by racial/ethnic groups, the model included both survey wave and race/ethnicity, along with their interaction term. Potential confounding characteristics were selected based on conceptual relevance and statistical significance, with the final model adjusting for age at diagnosis, years since diagnosis, marital status, insurance type, and education level. Three sensitivity analyses were conducted: (1) modelling social isolation and stress scores separately; (2) applying inverse probability weighting for non‐response; (3) imputing missing data in household income level with both individual‐ and neighbourhood‐level SES characteristics. Statistical analyses were conducted in STATA 17.0 (StataCorp, College Station, TX).

## 3 Results

In total, the study received responses from 1899 BCS, with the median time from diagnosis to first survey being 5.1 years (IQR: 2.3–9.2). Of the responders, 1317 (69.4%) were non‐Hispanic White (‘White’), 450 (23.7%) were non‐Hispanic Black (‘Black’), 53 (2.8%) were Hispanic, 75 (4.0%) were Asian and 2 (0.1%) were Native Americans. Limited by sample sizes, further analyses on racial disparities focused on comparing between Black and White BCS. Compared to White BCS (Table 1), Black BCS were significantly more likely to be diagnosed at older ages, with triple‐negative breast cancer, at later stages, and with higher comorbidity burden (all *p* < 0.05). In terms of SES, significantly more Black BCS were single, on Medicaid, and had lower levels of education and household income (all *p* < 0.001); they were also significantly more likely to live in neighbourhoods with lower income and education level, which was also captured by more disadvantaged neighbourhood ADIs (all *p* < 0.001).

**TABLE 1:** Characteristics of the survey respondents stratified by racial/ethnic groups.

Factor, no. (%)	White BCS (*n* = 1317)	Black BCS (*n* = 450)	Others^a^ (*n* = 132)	*p* ^b^
Age at breast cancer diagnosis, mean (SD)	54.3 (11.5)	55.8 (12.8)	48.2 (11.4)	0.036
Years from diagnosis to first survey, median (IQR)	5.2 (2.2, 9.2)	5.4 (2.6, 9.3)	4.3 (2.1, 8.1)	0.25
Charlson comorbidity index				0.001
0	1116 (88.7)	354 (81.9)	111 (88.8)	
1	62 (4.9)	30 (6.9)	7 (5.6)	
≥ 2	80 (6.4)	48 (11.1)	7 (5.6)	
Breast cancer subtype				< 0.001
HR+/HER2−	688 (70.8)	188 (57.0)	62 (63.3)	
HR+/HER2+	103 (10.6)	31 (9.4)	18 (18.4)	
HR‐/HER2+	45 (4.6)	30 (9.1)	3 (3.1)	
TNBC	136 (14.0)	81 (24.5)	15 (15.3)	
Tumour stage				0.005
0	210 (16.9)	89 (20.8)	24 (19.7)	
I	597 (48.2)	163 (38.2)	50 (41.0)	
II	305 (24.6)	123 (28.8)	36 (29.5)	
III‐IV	127 (10.3)	52 (12.2)	12 (9.8)	
Insurance type				< 0.001
Private insurance	958 (72.7)	221 (49.1)	104 (78.8)	
Medicare	246 (18.7)	113 (25.1)	12 (9.1)	
Medicaid	19 (1.4)	81 (18.0)	6 (4.5)	
Others	94 (7.1)	35 (7.8)	10 (7.6)	
Education level				< 0.001
Did not finish high school	6 (0.5)	11 (3.0)	1 (0.8)	
High school graduate or equivalent	107 (8.9)	43 (11.6)	12 (9.8)	
Trade/technical school, some college, Associate's	249 (20.7)	130 (34.9)	15 (12.2)	
Bachelor's degree	349 (29.0)	84 (22.6)	42 (34.1)	
Graduate or professional degree	493 (40.9)	104 (28.0)	53 (43.1)	
Marital status				< 0.001
Single/never married	82 (6.3)	124 (28.2)	17 (13.2)	
Married/living with a partner	966 (74.7)	172 (39.2)	93 (72.1)	
Divorced, separated, or widowed	245 (18.9)	143 (32.6)	19 (14.7)	
No. of people living together, median (IQR)	1.0 (1.0, 2.0)	1.0 (1.0, 2.0)	2.0 (1.0, 3.0)	0.25
Annual household income				< 0.001
< $35,000	35 (5.3)	68 (30.6)	13 (18.1)	
$35,000–$99,999	217 (33.0)	116 (52.3)	24 (33.3)	
$100,000–$199,999	251 (38.1)	32 (14.4)	23 (31.9)	
≥ $200,000	155 (23.6)	6 (2.7)	12 (16.7)	
Missing or prefer not to answer^c^	659	228	60	
Annual household income of neighbourhood, mean (SD)	103,918 (46,788)	62,355 (33,212)	94,241 (44,978)	< 0.001
% of households with annual income less than $30,000 in neighbourhood				< 0.001
< 10	558 (43.9)	58 (13.7)	47 (37.0)	
10–20	391 (30.7)	85 (20.0)	40 (31.5)	
20–40	271 (21.3)	167 (39.4)	29 (22.8)	
≥ 40	52 (4.1)	114 (26.9)	11 (8.7)	
% of adult population without a high school degree in neighbourhood				< 0.001
< 1	296 (23.3)	40 (9.4)	22 (17.3)	
1–5	532 (41.8)	101 (23.8)	47 (37.0)	
5–10	260 (20.4)	105 (24.8)	24 (18.9)	
≥ 10	184 (14.5)	178 (42.0)	34 (26.8)	
Area deprivation index (national ranking percentiles) of neighbourhood^d^				< 0.001
1^st^ quartile	528 (41.8)	28 (6.7)	43 (34.1)	
2^nd^ quartile	461 (36.5)	78 (18.5)	50 (39.7)	
3^rd^ quartile	213 (16.9)	185 (43.9)	23 (18.3)	
4^th^ quartile (most disadvantaged)	61 (4.8)	130 (30.9)	10 (7.9)	

^a^ Other BCS include 75 Asian BCS, 53 Hispanic BCS and 2 Native American BCS.

^b^ *p* values for the comparison between White and Black BCS were estimated using standard descriptive statistics described in Methods. The comparison of neighbourhood‐level characteristics were adjusted for clusters in census block group.

^c^ Household income was only asked in one wave of survey and some preferred not to answer, resulting in large numbers of missing data. Missing values for other variables were minimal, so their missing categories were not shown.

^d^ ADI national ranking percentiles were constructed by ranking the ADI from low to high for the nation and grouping them into bins corresponding to each 1% range of the ADI, with those in the hundredth being the most disadvantaged.

**FIGURE 1:**
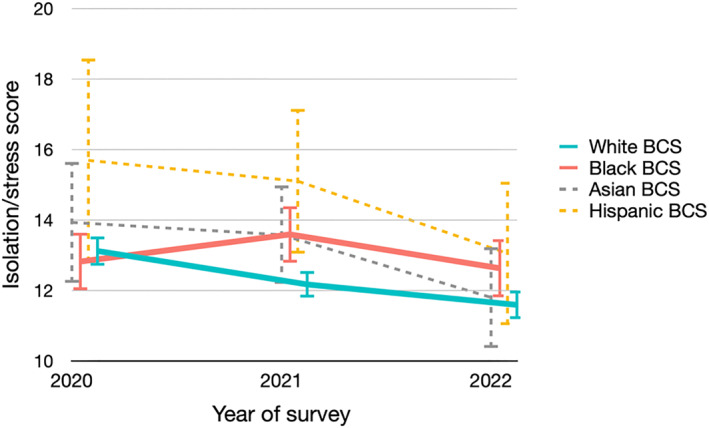
Isolation/stress scores in three waves of surveys by racial/ethnic groups.

Overall, the levels of stress and isolation were moderate (Figure 1). For White BCS, the average isolation/stress score continuously improved from 2020 to 2022, declining from 13.1 to 12.2 to 11.6 (*P*‐trend < 0.001) (Supporting Information S1: Table 1). The average scores also declined for Asian (from 13.9 to 13.6 to 11.8) and Hispanic BCS (from 15.7 to 15.1 to 13.1), though the sample sizes limited the power to detect significant trends. However, this positive trend was not observed for Black BCS, with the average score in the three waves of surveys being 12.8, 13.6 and 12.6, respectively (*P*‐trend = 0.84). To better understand where the disparities lay, we examined the responses to the individual questions (Supporting Information S1: Table 2). Compared to Black BCS, White BCS were significantly more likely to feel confident in getting medical help and keeping up with work and home responsibilities in the 2021 and 2022 survey (all *p* < 0.005). Meanwhile, Black BCS were significantly more likely to feel isolated, overwhelmed, and worried about getting sick in 2021, and more likely to worry about going to hospitals in 2022 (all *p* < 0.05).

The figure presented the average isolation/stress scores and the corresponding 95% CIs across the three survey waves, stratified by racial/ethnic groups. Detailed values are provided in Supporting Information S1: Table 1.

To account for the repeated measures within individuals, we applied mixed‐effects models to examine the changes in the isolation/stress score over time for different racial/ethnic groups (Table 2). Our findings suggested that the isolation stress score significantly decreased each year for White BCS (both *p* < 0.001); while for Black BCS, there was only a significant decrease in the last wave of survey (−0.81; 95% CI: −1.48 to −0.15; *p* = 0.016). Compared to White BCS, Black BCS had significantly higher estimated scores in 2021 (1.34; 95% CI: 0.57 to 2.10; *p* = 0.001) and 2022 (1.14; 95% CI: 0.34 to 1.95; *p* = 0.005). The racial differences observed in the last 2 years became non‐significant after adjusting for marital status, insurance type and education level. To be specific, BCS who were single, covered by Medicaid and without a high school degree had significantly higher isolation/stress scores (all *p* < 0.005). The findings remained consistent in the sensitivity analysis using inverse probability weighting to account for non‐response (Supporting Information S1: Table 3). In additional analysis adjusting for household income, both complete‐case analysis and multiple imputation showed that BCS with lower income levels experienced significantly higher isolation/stress scores (both *p* < 0.005) (Supporting Information S1: Table 4).

**TABLE 2:** Mixed‐effects models of total isolation/stress score with interaction between survey year and racial/ethnic group.

	Model 1: Racial disparities over time				Model 2: Racial disparities over time further adjusting for potential confounders			
	Coefficient	S.E.	95% CI	*p*	Coefficient	S.E.	95% CI	*p*
Racial/ethnic group in 2020 (ref: White BCS)								
Black BCS	−0.29	0.39	[−1.06, 0.48]	0.46	−0.94	0.47	[−1.87, −0.013]	0.047
Others	1.72	0.66	[0.43, 3.00]	0.009	1.04	0.69	[−0.32, 2.40]	0.13
Survey year in white BCS (ref: 2020)								
2021	−1.10	0.18	[−1.45, −0.74]	< 0.001	−1.11	0.19	[−1.48, −0.74]	< 0.001
2022	−1.72	0.18	[−2.08, −1.36]	< 0.001	−1.70	0.19	[−2.08, −1.32]	< 0.001
Racial/ethnic group × survey year interaction (ref: White BCS × 2020)								
Black BCS × 2021	1.63	0.39	[0.87, 2.39]	< 0.001	1.33	0.41	[0.52, 2.13]	0.001
Black BCS × 2022	1.43	0.40	[0.65, 2.22]	< 0.001	1.22	0.42	[0.39, 2.05]	0.004
Others × 2021	0.24	0.61	[−0.95, 1.43]	0.69	0.41	0.63	[−0.82, 1.64]	0.51
Others × 2022	−0.69	0.61	[−1.89, 0.50]	0.26	−0.61	0.63	[−1.84, 0.63]	0.34
Age at breast cancer diagnosis, per 5‐year increase					−0.45	0.083	[−0.61, −0.29]	< 0.001
Years from diagnosis to survey, per 1‐year increase					−0.02	0.031	[−0.08, 0.04]	0.48
Marital status (ref: married/living with partner)								
Single/never married					1.56	0.52	[0.55, 2.57]	0.002
Divorced, separated, or widowed					0.61	0.38	[−0.14, 1.36]	0.11
Insurance type (ref: private insurance)								
Medicare					1.06	0.49	[0.10, 2.02]	0.030
Medicaid					2.81	0.72	[1.39, 4.23]	< 0.001
Others					0.42	0.98	[−1.50, 2.33]	0.67
Education level (ref: graduate/professional degree)								
Bachelor's degree					0.16	0.36	[−0.55, 0.87]	0.66
Trade/technical school, some college, Associate's					−0.3	0.39	[−1.06, 0.47]	0.45
High school graduate/GED					−0.77	0.52	[−1.79, 0.26]	0.14
Did not finish high school					5.00	1.49	[2.07, 7.93]	0.001

*Note:* Model one included survey year, racial/ethnic group, and their interaction to examine racial differences in the change of total score across survey years. The model employed random intercepts for participants to account for within‐person correlation due to repeated measurements, and the other covariates were included as fixed effects. Model 2 further adjusted for age at diagnosis, years since diagnosis, marital status, insurance type, and education level. The socioeconomic covariates were selected if they had a statistically significant association with the outcome in univariate analyses. No significant associations were found between the isolation/stress score and the clinical and tumour characteristics, so they were not further adjusted in the multivariate model.

Abbreviations: BCS, breast cancer survivors; CI, confidence interval; GED, General Educational Diploma; ref, reference; S.E., standard error.

We conducted further sensitivity analysis by examining the two subscales: the social isolation score (Supporting Information S1: Table 5) and the stress score (Supporting Information S1: Table 6). The sensitivity analysis revealed similar patterns, with significant declines over time observed only among White BCS (all *p* < 0.001). After further adjusting for SES factors, we found that the social isolation score was significantly associated with family composition, being lower for BCS who were married compared to those who were single (−0.95; 95% CI: −1.44 to −0.46; *p* < 0.001) and decreased by 0.12 for each additional household member living together (95% CI: −0.23 to −0.02, *p* = 0.026). Whereas for the stress score, it was significantly higher for BCS who did not finish high school and for BCS who were on Medicaid (both *p* < 0.001). The racial differences observed in both scores were no longer statistically significant after adjusting for the corresponding SES factors.

## 4 Discussion

This three‐wave longitudinal survey study including 1899 BCS from 2020 to 2022 examined changes in stress and isolation levels over time, and found disparate patterns across racial/ethnic groups. Among White BCS, isolation and stress levels showed a steady decline across the three survey waves. However, this positive trend was not observed among Black BCS. Compared with White BCS, Black BCS experienced significantly higher levels of isolation and stress in 2021 and 2022. The isolation/stress score only started declining for Black BCS in the last wave of survey. Our findings are consistent with prior longitudinal research in the general population, suggesting that racial/ethnic minorities may experience more persistent distress and slower recovery as the pandemic subsided (Patler and Langer 2024). In contrast, a recent large‐scale study of cancer survivors based on early‐pandemic, cross‐sectional data reported no racial disparities in mental health (Qian et al. 2025). Differences in study design and timing may explain these contrasting findings, highlighting how our longitudinal approach offered additional insight in understanding how disparities may evolve over time.

Further understanding the stressors associated with these disparities is important in informing potential interventions. In our survivor cohort, their isolation/stress levels were not associated with their clinical characteristics at diagnosis, while the observed racial differences may be explained by the varying distributions of SES factors across different survivor groups. To be specific, BCS who had lower education, lower household income and/or covered by Medicaid had significantly higher isolation/stress scores. Our findings indicate that survivors with limited SES resources may be more vulnerable to the challenges posed by the pandemic, especially financial hardships and economic insecurity (Brivio et al. 2020; Matsubayashi et al. 2022; Zamanzadeh et al. 2024). In the event of future crises, we should consider providing targeted support to these vulnerable populations, including financial assistance programs, affordable mental health services, and improved access through integrating telehealth technologies (Yabroff et al. 2020). In addition, policies should also aim to reduce the feelings of uncertainty about future financial stability, as prior research suggested that expected income loss may have a stronger impact on mental wellbeing (Zamanzadeh et al. 2024). These findings also emphasise the importance of building resilience in disproportionately affected communities through initiatives like educational programs and community‐based support networks (Howell et al. 2008; la Cour and Cutchin 2013).

Upon further examination of factors associated with the social isolation score, we found that family composition played an important role, with BCS who were married or living with more people together reporting significantly lower levels of social isolation. This finding is consistent with previous research emphasising the importance of social support from family members in mitigating feelings of isolation (Brivio et al. 2020; Miaskowski et al. 2021; Zaken et al. 2022). In addition to the reduced level of social support from family and friends, patients might have also experienced a substantial loss of support received from healthcare providers and other cancer survivors (Haase et al. 2021; Savard et al. 2021). Thus, increasing opportunities for patients and survivors to connect with healthcare providers and one another, such as organising workshops or online forums, appear to be promising ways to strengthen their social support network, thereby enhancing their resilience to stress (Howell et al. 2008).

The major strength of our study is its longitudinal design, enabling us to track the changes in the isolation and stress level of a large group of BCS throughout the pandemic. In contrast, most of the previous studies among cancer survivors were cross‐sectional and/or conducted in the early phases of the pandemic (Almeida et al. 2024; Muls et al. 2022; Zhang et al. 2022), limiting our understanding of the differential experiences of BCS as the pandemic evolved over time. As all three waves of surveys were conducted from July to September, any seasonal effects were controlled effectively. Furthermore, through utilising individual SES data and geocoding to acquire neighbourhood‐level SES data, we were able to better understand the underlying factors associated with the racial disparities observed and provide actionable insights for interventions.

Despite these strengths, there were several limitations to be noted. First, as a single‐institution study, our patient cohort was not nationally representative (e.g., had relatively higher levels of education and income). In addition, the overall response rate of the survey was 62.8%, raising concerns of selection bias. It is plausible that those experiencing higher levels of distress may be less likely to respond, potentially leading to an underestimation of the overall stress and isolation levels. To address this, we conducted sensitivity analysis using inverse probability weighting, and the major findings remained consistent. Secondly, as there were no existing instruments to measure COVID‐associated isolation and stress when the first wave of survey was conducted, the measurement we used differed from those used in other studies, limiting our ability to make direct comparisons externally. This also meant that the study lacked baseline isolation/stress levels measured before the pandemic, limiting our ability to examine whether the isolation/stress level has recovered. Nevertheless, the 11‐item score we employed showed great internal consistency across all three waves of survey. Finally, the small sample sizes of Asian and Hispanic BCS in the study limited the statistical power to identify any meaningful patterns, and we were unable to robustly examine their experiences as the pandemic subsided in our analysis.

## 5 Conclusions

In conclusion, our three‐wave longitudinal study of 1899 BCS revealed that, despite demonstrating considerable resilience in general, the recovery of mental health might follow different trajectories for BCS from different racial/ethnic groups. In our cohort, only White BCS experienced significant improvements in levels of isolation and stress over time, while such positive trend was not observed among Black BCS. Mixed‐effects modelling found that these disparities were associated with disparities in SES, including insurance coverage, education level, household income and family composition. Our findings underscore the need for developing targeted interventions and support systems for these vulnerable populations. Future research should continue tracking mental health recovery over time, particularly among more diverse racial and ethnic groups, and evaluate the effectiveness of tailored interventions in reducing these disparities, in order to build more equitable survivorship care systems.

## Author Contributions

Study conception and design were performed by F.Z., O.I.O. and D.H. Data collection and formal analysis were performed by F.Z. and D.H. The first draft of the manuscript was written by F.Z. and all authors (F.Z., J.Q.F., N.J., G.F.F., R.N., D.S.L., O.I.O. and D.H.) commented on previous versions of the manuscript. All authors (F.Z., J.Q.F., N.J., G.F.F., R.N., D.S.L., O.I.O. and D.H.) read and approved the final manuscript.

## Ethics Statement

This study was approved by the University of Chicago Institutional Review Board in accordance with the Declaration of Helsinki.

## Consent

All participants provided their written informed consent.

## Conflicts of Interest

The authors declare no conflicts of interest.

## Permission to Reproduce Material From Other Sources

The authors have nothing to report.

## Supporting information

Supporting Information S1

## Data Availability

The dataset analysed in the current study is not publicly available as required by the study protocol but can be made available from the corresponding author on reasonable request under the instruction of the University of Chicago Institutional Review Board.
