# Supplementary material for: Longitudinal Changes in Stress and Isolation Among Multi-Ethnic Breast Cancer Survivors Throughout COVID-19
Source: Stress Health. Author manuscript; Available in PMC 2025 Jul 9. (PMC12175489; doi:10.1002/smi.70063)
Supplement: Supporting Information S1 [file NIHMS2091486-supplement-Supporting_Information_S1.docx]

**Supplemental Table 1.** Average Isolation/Stress Scores and 95% Confidence Intervals by Racial/Ethnic Group and Survey Year

| **Average Isolation/Stress Score (95% CI)** | **2020 Survey** | **2021 Survey** | **2022 Survey** | ***P*-trend** |
| --- | --- | --- | --- | --- |
| **Racial/Ethnic Group** |  |  |  |  |
| White BCS | 13.12 (12.71, 13.53) | 12.17 (11.79, 12.55) | 11.59 (11.19, 11.99) | < .001 |
| Black BCS | 12.82 (12.00, 13.64) | 13.59 (12.79, 14.39) | 12.63 (11.81, 13.45) | .84 |
| Asian BCS | 13.93 (12.22, 15.64) | 13.58 (12.18, 14.98) | 11.80 (10.37, 13.23) | .044 |
| Hispanic BCS | 15.69 (12.80, 18.58) | 15.10 (13.05, 17.15) | 13.05 (11.02, 15.08) | .29 |

**Supplemental Table 2.** Responses to the 11 Isolation/Stress Questions in the Three Waves of Surveys among Black and White BCS

| **Question, No. (%)** | **Wave 1 (2020) ^a^** | | | **Wave 2 (2021)** | | | **Wave 3 (2022)** | | |
| --- | --- | --- | --- | --- | --- | --- | --- | --- | --- |
|  | **White BCS**  **(n = 937)** | **Black BCS**  **(n = 294)** | ***P* ^b^** | **White BCS**  **(n = 947)** | **Black BCS**  **(n = 308)** | ***P*** | **White BCS**  **(n = 895)** | **Black BCS**  **(n = 247)** | ***P*** |
| **How isolated are you feeling now due to COVID-19?** |  |  | .13 |  |  | <.001 |  |  | .12 |
| Not at all | 111 (11.9) | 64 (22.2) |  | 359 (38.5) | 89 (29.9) |  | 534 (60.0) | 137 (55.7) |  |
| A little | 223 (23.9) | 59 (20.5) |  | 326 (34.9) | 68 (22.8) |  | 207 (23.3) | 52 (21.1) |  |
| Somewhat | 367 (39.4) | 86 (29.9) |  | 190 (20.4) | 102 (34.2) |  | 110 (12.4) | 44 (17.9) |  |
| Very | 207 (22.2) | 62 (21.5) |  | 48 (5.1) | 32 (10.7) |  | 32 (3.6) | 11 (4.5) |  |
| Extremely | 24 (2.6) | 17 (5.9) |  | 10 (1.1) | 7 (2.3) |  | 7 (0.8) | 2 (0.8) |  |
| **Do you feel supported by friends and family during this time of COVID-19?* ^c^** |  |  | .43 |  |  | .98 |  |  | .14 |
| Not at all | 17 (1.8) | 4 (1.4) |  | 13 (1.4) | 6 (2.0) |  | 36 (4.0) | 6 (2.4) |  |
| A little | 18 (1.9) | 5 (1.7) |  | 29 (3.1) | 12 (4.0) |  | 28 (3.1) | 9 (3.7) |  |
| Somewhat | 98 (10.5) | 37 (12.8) |  | 123 (13.2) | 41 (13.8) |  | 122 (13.7) | 39 (15.9) |  |
| Very | 312 (33.6) | 81 (28.0) |  | 437 (46.8) | 127 (42.8) |  | 361 (40.4) | 112 (45.7) |  |
| Extremely | 484 (52.1) | 162 (56.1) |  | 332 (35.5) | 111 (37.4) |  | 346 (38.7) | 79 (32.2) |  |
| **How optimistic are you that you are supported to manage life’s challenges successfully?*** |  |  | .33 |  |  | .074 |  |  | .82 |
| Not at all | 12 (1.3) | 3 (1.0) |  | 6 (0.6) | 7 (2.3) |  | 24 (2.7) | 7 (2.9) |  |
| A little | 15 (1.6) | 8 (2.8) |  | 21 (2.3) | 6 (2.0) |  | 27 (3.1) | 6 (2.5) |  |
| Somewhat | 127 (13.7) | 38 (13.2) |  | 143 (15.3) | 65 (21.8) |  | 154 (17.4) | 46 (18.9) |  |
| Very | 417 (45.1) | 115 (40.1) |  | 468 (50.2) | 129 (43.3) |  | 417 (47.2) | 112 (46.1) |  |
| Extremely | 353 (38.2) | 123 (42.9) |  | 294 (31.5) | 91 (30.5) |  | 262 (29.6) | 72 (29.6) |  |
| **If you need medical help, how confident are you that you can find someone to take you to the doctor's office?*** |  |  | .053 |  |  | <.001 |  |  | .0012 |
| Not at all | 15 (1.6) | 3 (1.0) |  | 18 (1.9) | 6 (2.0) |  | 28 (3.1) | 10 (4.0) |  |
| A little | 15 (1.6) | 7 (2.4) |  | 13 (1.4) | 10 (3.3) |  | 18 (2.0) | 7 (2.8) |  |
| Somewhat | 39 (4.2) | 26 (8.9) |  | 54 (5.8) | 30 (10.0) |  | 55 (6.2) | 25 (10.1) |  |
| Very | 186 (20.2) | 60 (20.5) |  | 334 (35.8) | 121 (40.5) |  | 299 (33.5) | 95 (38.5) |  |
| Extremely | 666 (72.3) | 196 (67.1) |  | 514 (55.1) | 132 (44.1) |  | 492 (55.2) | 110 (44.5) |  |
| **Did you feel you could keep up with your work and home responsibilities (including working from home now)?*** |  |  | .038 |  |  | <.001 |  |  | <.001 |
| Never | 9 (1.0) | 12 (4.3) |  | 5 (0.5) | 2 (0.7) |  | 2 (0.2) | 5 (2.1) |  |
| Rarely | 39 (4.2) | 18 (6.5) |  | 16 (1.7) | 18 (6.1) |  | 24 (2.7) | 4 (1.7) |  |
| Sometimes | 132 (14.3) | 45 (16.2) |  | 133 (14.5) | 67 (22.9) |  | 116 (13.3) | 61 (25.2) |  |
| Often | 284 (30.9) | 75 (27.1) |  | 337 (36.7) | 86 (29.4) |  | 311 (35.5) | 76 (31.4) |  |
| Always | 456 (49.6) | 127 (45.8) |  | 428 (46.6) | 120 (41.0) |  | 422 (48.2) | 96 (39.7) |  |
| **Have you felt fearful?** |  |  | .0045 |  |  | .50 |  |  | .93 |
| Never | 232 (25.1) | 103 (36.4) |  | 194 (20.9) | 89 (30.3) |  | 238 (26.8) | 83 (33.7) |  |
| Rarely | 266 (28.7) | 69 (24.4) |  | 336 (36.2) | 77 (26.2) |  | 348 (39.2) | 65 (26.4) |  |
| Sometimes | 322 (34.8) | 81 (28.6) |  | 328 (35.4) | 91 (31.0) |  | 248 (28.0) | 79 (32.1) |  |
| Often | 96 (10.4) | 21 (7.4) |  | 62 (6.7) | 27 (9.2) |  | 43 (4.8) | 13 (5.3) |  |
| Always | 10 (1.1) | 9 (3.2) |  | 7 (0.8) | 10 (3.4) |  | 10 (1.1) | 6 (2.4) |  |
| **Have your worries overwhelmed you?** |  |  | .55 |  |  | .0092 |  |  | .38 |
| Never | 302 (32.5) | 104 (36.9) |  | 296 (31.9) | 83 (28.2) |  | 266 (30.1) | 84 (34.3) |  |
| Rarely | 332 (35.8) | 73 (25.9) |  | 345 (37.2) | 92 (31.3) |  | 344 (39.0) | 66 (26.9) |  |
| Sometimes | 222 (23.9) | 67 (23.8) |  | 237 (25.6) | 94 (32.0) |  | 225 (25.5) | 70 (28.6) |  |
| Often | 60 (6.5) | 29 (10.3) |  | 39 (4.2) | 21 (7.1) |  | 43 (4.9) | 20 (8.2) |  |
| Always | 12 (1.3) | 9 (3.2) |  | 10 (1.1) | 4 (1.4) |  | 5 (0.6) | 5 (2.0) |  |
| **How much did you worry about getting sick from COVID-19?** |  |  | .12 |  |  | .018 |  |  | .12 |
| Never | 62 (6.7) | 36 (12.8) |  | 65 (7.0) | 32 (10.8) |  | 96 (10.8) | 38 (15.5) |  |
| Rarely | 247 (26.7) | 60 (21.4) |  | 251 (27.1) | 65 (22.0) |  | 269 (30.4) | 56 (22.9) |  |
| Sometimes | 419 (45.3) | 94 (33.5) |  | 409 (44.1) | 98 (33.1) |  | 335 (37.8) | 78 (31.8) |  |
| Often | 153 (16.6) | 59 (21.0) |  | 158 (17.0) | 67 (22.6) |  | 151 (17.0) | 51 (20.8) |  |
| Always | 43 (4.7) | 32 (11.4) |  | 44 (4.7) | 34 (11.5) |  | 35 (4.0) | 22 (9.0) |  |
| **How often were you worried that you might need to go to the hospital?** |  |  | .16 |  |  | .053 |  |  | .0016 |
| Never | 283 (30.7) | 100 (35.6) |  | 285 (31.0) | 86 (29.3) |  | 340 (38.7) | 72 (29.5) |  |
| Rarely | 341 (36.9) | 100 (35.6) |  | 377 (41.0) | 106 (36.1) |  | 349 (39.7) | 99 (40.6) |  |
| Sometimes | 220 (23.8) | 56 (19.9) |  | 180 (19.6) | 60 (20.4) |  | 133 (15.1) | 51 (20.9) |  |
| Often | 64 (6.9) | 16 (5.7) |  | 63 (6.8) | 27 (9.2) |  | 40 (4.6) | 16 (6.6) |  |
| Always | 15 (1.6) | 9 (3.2) |  | 15 (1.6) | 15 (5.1) |  | 16 (1.8) | 6 (2.5) |  |
| **Lately, have you felt hopeful?*** |  |  | <.001 |  |  | .67 |  |  | .97 |
| Never | 7 (0.8) | 11 (3.9) |  | 19 (2.0) | 9 (3.0) |  | 6 (0.7) | 12 (4.9) |  |
| Rarely | 74 (8.0) | 13 (4.6) |  | 29 (3.1) | 13 (4.4) |  | 28 (3.2) | 9 (3.7) |  |
| Sometimes | 291 (31.3) | 67 (23.5) |  | 194 (20.8) | 76 (25.7) |  | 190 (21.5) | 55 (22.4) |  |
| Often | 377 (40.5) | 88 (30.9) |  | 469 (50.3) | 111 (37.5) |  | 428 (48.5) | 88 (35.9) |  |
| Always | 181 (19.5) | 106 (37.2) |  | 222 (23.8) | 87 (29.4) |  | 231 (26.2) | 81 (33.1) |  |
| **In the past month, to what extent have you felt nervous or stressed?** |  |  | .0085 |  |  | .37 |  |  | .21 |
| Never | 48 (5.2) | 37 (12.9) |  | 70 (7.5) | 42 (14.2) |  | 53 (6.0) | 31 (12.6) |  |
| Rarely | 231 (24.8) | 71 (24.8) |  | 296 (31.7) | 76 (25.7) |  | 281 (31.7) | 67 (27.2) |  |
| Sometimes | 410 (44.1) | 114 (39.9) |  | 400 (42.8) | 125 (42.2) |  | 392 (44.2) | 106 (43.1) |  |
| Often | 210 (22.6) | 51 (17.8) |  | 148 (15.8) | 48 (16.2) |  | 145 (16.4) | 39 (15.9) |  |
| Always | 31 (3.3) | 13 (4.5) |  | 20 (2.1) | 5 (1.7) |  | 15 (1.7) | 3 (1.2) |  |
| ^a^ Slightly few patients (930 Whites and 291 Black BCS) were reported in our previous publication (Zhao et al. Cancer. 2021;127:4072-4080), as several BCS responded to the first wave of survey after the cut-off date we chose during the initial analysis.  ^b^ *P-*values for the comparison between White and Black BCS were calculated using Wilcoxon Rank-Sum tests.  ^c^ To ensure that higher scores reflected greater levels of stress and isolation, five positively phrased items (marked with asterisk*) were reversely coded before summing them with the remaining items when calculating the total isolation/stress score. | | | | | | | | | |

**Supplemental Table 3.** Mixed-effects Models of Total Isolation/Stress Score with Interaction between Survey Year and Racial/Ethnic Group Weighted for Non-Response

|  | **Model 1W: Racial Disparities over Time Weighted for Non-Response** | | | | **Model 2W: Racial Disparities over Time Further Adjusting for Potential Confounders**  **Weighted for Non-Response** | | | |
| --- | --- | --- | --- | --- | --- | --- | --- | --- |
|  | **Coefficient** | **S.E.** | **95% CI** | ***P*** | **Coefficient** | **S.E.** | **95% CI** | ***P*** |
| **Racial/ethnic group (ref: White BCS)** |  |  |  |  |  |  |  |  |
| Black BCS | -0.29 | 0.44 | [-1.15, 0.57] | .50 | -0.96 | 0.54 | [-2.02, 0.09] | .072 |
| Others | 1.94 | 0.73 | [0.50, 3.38] | .008 | 1.32 | 0.82 | [-0.29, 2.92] | .108 |
| **Survey year (ref: 2020)** |  |  |  |  |  |  |  |  |
| 2021 | -1.07 | 0.18 | [-1.42, -0.72] | < .001 | -1.08 | 0.19 | [-1.44, -0.71] | < .001 |
| 2022 | -1.70 | 0.19 | [-2.07, -1.32] | < .001 | -1.68 | 0.21 | [-2.08, -1.27] | < .001 |
| **Racial/ethnic group × Survey year interaction**  **(ref: White BCS × 2020)** |  |  |  |  |  |  |  |  |
| Black BCS × 2021 | 1.53 | 0.42 | [0.71, 2.34] | < .001 | 1.24 | 0.45 | [0.37, 2.11] | .005 |
| Black BCS × 2022 | 1.39 | 0.43 | [0.54, 2.24] | .001 | 1.19 | 0.45 | [0.30, 2.07] | .009 |
| Others × 2021 | 0.10 | 0.58 | [-1.02, 1.23] | .86 | 0.27 | 0.60 | [-0.90, 1.44] | .65 |
| Others × 2022 | -0.76 | 0.60 | [-1.95, 0.42] | .21 | -0.67 | 0.64 | [-1.92, 0.58] | .29 |
| **Age at breast cancer diagnosis,**  **per 5-year increase** |  |  |  |  | -0.45 | 0.083 | [-0.61, -0.29] | < .001 |
| **Years from diagnosis to survey,**  **per 1-year increase** |  |  |  |  | -0.02 | 0.03 | [-0.09, 0.04] | .44 |
| **Marital status (ref: married/living with partner)** |  |  |  |  |  |  |  |  |
| Single/never married |  |  |  |  | 1.52 | 0.56 | [0.42, 2.61] | .007 |
| Divorced, separated, or widowed |  |  |  |  | 0.5 | 0.42 | [-0.33, 1.33] | .24 |
| **Insurance type (ref: private insurance)** |  |  |  |  |  |  |  |  |
| Medicare |  |  |  |  | 1.13 | 0.5 | [0.16, 2.11] | .022 |
| Medicaid |  |  |  |  | 2.84 | 0.79 | [1.28, 4.39] | < .001 |
| Others |  |  |  |  | 0.45 | 0.79 | [-1.10, 1.99] | .57 |
| **Education level (ref: graduate/professional degree)** |  |  |  |  |  |  |  |  |
| Bachelor’s degree |  |  |  |  | 0.19 | 0.37 | [-0.54, 0.92] | .61 |
| Trade/technical school, some college, Associate’s |  |  |  |  | -0.09 | 0.39 | [-0.87, 0.68] | .81 |
| High school graduate/GED |  |  |  |  | -0.67 | 0.57 | [-1.78, 0.44] | .24 |
| Did not finish high school |  |  |  |  | 4.98 | 1.51 | [2.02, 7.94] | .001 |
| Abbreviations: S.E., standard error; CI, confidence interval; ref, reference; BCS, breast cancer survivors; GED, General Educational Diploma.  Models 1W and 2W were set up equivantly with Models 1 and 2 in Table 2, but estimated using inverse probability weighting to account for non-response. The probability of non-response was modeled using logistic regression across the full cohort, with race/ethnicity and year of enrollment included as predictors, as both were significantly associated with non-response. | | | | | | | | |

**Supplemental Table 4.** Mixed-effects Models of Total Isolation/Stress Score with Interaction between Survey Year and Racial/Ethnic Group, including Household Income with/without Multiple Imputation

|  | **Model 3: Multivariate Model**  **in Complete Case Analysis** | | | | **Model 3I: Multivariate Model**  **in Imputed Dataset** | | | |
| --- | --- | --- | --- | --- | --- | --- | --- | --- |
|  | **Coefficient** | **S.E.** | **95% CI** | ***P*** | **Coefficient** | **S.E.** | **95% CI** | ***P*** |
| **Racial/ethnic group (ref: White BCS)** |  |  |  |  |  |  |  |  |
| Black BCS | -1.74 | 0.63 | [-2.98, -0.50] | .006 | -1.43 | 0.52 | [-2.46, -0.39] | .007 |
| Others | 0.84 | 0.94 | [-1.00, 2.68] | .37 | 0.89 | 0.67 | [-0.43, 2.21] | .19 |
| **Survey year (ref: 2020)** |  |  |  |  |  |  |  |  |
| 2021 | -1.06 | 0.23 | [-1.51, -0.60] | < .001 | -1.15 | 0.17 | [-1.50, -0.81] | < .001 |
| 2022 | -1.81 | 0.25 | [-2.29, -1.33] | < .001 | -1.70 | 0.20 | [-2.10, -1.31] | < .001 |
| **Racial/ethnic group × Survey year interaction**  **(ref: White BCS × 2020)** |  |  |  |  |  |  |  |  |
| Black BCS × 2021 | 1.51 | 0.49 | [0.54, 2.47] | .002 | 1.51 | 0.46 | [0.59, 2.42] | .002 |
| Black BCS × 2022 | 1.47 | 0.54 | [0.42, 2.53] | .006 | 1.62 | 0.51 | [0.61, 2.64] | .002 |
| Others × 2021 | -0.02 | 0.79 | [-1.56, 1.52] | .98 | 0.31 | 0.60 | [-0.87, 1.49] | .61 |
| Others × 2022 | -0.43 | 0.84 | [-2.09, 1.22] | .61 | -0.82 | 0.63 | [-2.06, 0.42] | .19 |
| **Age at breast cancer diagnosis, per 5-year increase** | -0.35 | 0.11 | [-0.56, -0.13] | .002 | -0.49 | 0.08 | [-0.65, -0.32] | < .001 |
| **Years from diagnosis to survey,**  **per 1-year increase** | -0.03 | 0.04 | [-0.11, 0.05] | .42 | -0.03 | 0.03 | [-0.09, 0.03] | .35 |
| **Marital status (ref: married/living with partner)** |  |  |  |  |  |  |  |  |
| Single/never married | 1.36 | 0.69 | [0.01, 2.71] | .048 | 1.13 | 0.51 | [0.13, 2.12] | .027 |
| Divorced, separated, or widowed | 0.12 | 0.53 | [-0.92, 1.16] | .83 | 0.56 | 0.41 | [-0.24, 1.36] | .17 |
| **Insurance type (ref: private insurance)** |  |  |  |  |  |  |  |  |
| Medicare | 0.63 | 0.67 | [-0.69, 1.95] | .35 | 0.90 | 0.48 | [-0.04, 1.85] | .061 |
| Medicaid | 1.91 | 0.95 | [0.04, 3.77] | .045 | 1.52 | 0.80 | [-0.05, 3.09] | .057 |
| Others | 1.66 | 1.55 | [-1.38, 4.69] | .28 | 0.03 | 0.96 | [-1.84, 1.91] | .97 |
| **Education level (ref: graduate/professional degree)** |  |  |  |  |  |  |  |  |
| Bachelor’s degree | -0.45 | 0.48 | [-1.40, 0.49] | .35 | -0.15 | 0.36 | [-0.87, 0.56] | .67 |
| Trade/technical school, some college, Associate’s | -0.77 | 0.55 | [-1.84, 0.30] | .16 | -0.78 | 0.42 | [-1.61, 0.04] | .063 |
| High school graduate/GED | 1.60 | 0.72 | [0.19, 3.01] | .026 | 1.45 | 0.60 | [0.26, 2.64] | .017 |
| Did not finish high school | 1.23 | 2.15 | [-2.99, 5.44] | .57 | 2.62 | 1.68 | [-0.70, 5.93] | .12 |
| **Annual household income level (ref: ≥ $200,000)** |  |  |  |  |  |  |  |  |
| $100,000-$199,999 | 0.06 | 0.56 | [-1.04, 1.16] | .92 | 0.27 | 0.51 | [-0.75, 1.29] | .60 |
| $35,000-$99,999 | 0.20 | 0.61 | [-1.01, 1.40] | .75 | 0.76 | 0.62 | [-0.47, 2.00] | .22 |
| < $35,000 | 2.93 | 0.94 | [1.08, 4.77] | .002 | 2.86 | 0.89 | [1.10, 4.63] | .002 |
| Abbreviations: S.E., standard error; CI, confidence interval; ref, reference; BCS, breast cancer survivors; GED, General Educational Diploma.  Models 3 and 3I further adjusted for household income on the basis of Model 2 in Table 2, one as a complete case analysis and one using the imputed values. Multiple imputation was conducted employing both individual-level demographic and clinical characteristics as well as the neighborhood-level SES characteristics available in the study (as shown in Table 1), and imputed for the missing values in household income. We utilized the “mi” command in Stata, which imputes missing values while accounting for the longitudinal structure of the data. | | | | | | | | |

**Supplemental Table 5.** Mixed-effects Models of the **Social Isolation Score** with Interactions between Survey Year and Race/Ethnicity

|  | **Model 4: Racial Disparities over Time**  **in the Social Isolation Score** | | | | **Model 5: Racial Disparities over Time**  **in the Social Isolation Score**  **Further Adjusting for Potential Confounders** | | | |
| --- | --- | --- | --- | --- | --- | --- | --- | --- |
|  | **Coefficient** | **S.E.** | **95% CI** | ***P*** | **Coefficient** | **S.E.** | **95% CI** | ***P*** |
| **Racial/ethnic group (ref: White BCS)** |  |  |  |  |  |  |  |  |
| Black BCS | -0.10 | 0.17 | [-0.44, 0.24] | .57 | -0.37 | 0.22 | [-0.79, 0.06] | .090 |
| Others | 0.43 | 0.29 | [-0.15, 1.00] | .15 | 0.34 | 0.32 | [-0.28, 0.97] | .28 |
| **Survey year (ref: 2020)** |  |  |  |  |  |  |  |  |
| 2021 | -0.40 | 0.10 | [-0.59, -0.21] | < .001 | -0.46 | 0.11 | [-0.67, -0.24] | < .001 |
| 2022 | -0.51 | 0.10 | [-0.70, -0.32] | < .001 | -0.54 | 0.11 | [-0.75, -0.32] | < .001 |
| **Racial/ethnic group × Survey year interaction**  **(ref: White BCS × 2020)** |  |  |  |  |  |  |  |  |
| Black BCS × 2021 | 0.78 | 0.20 | [0.39, 1.18] | < .001 | 0.82 | 0.24 | [0.35, 1.29] | .001 |
| Black BCS × 2022 | 0.51 | 0.21 | [0.10, 0.92] | .015 | 0.36 | 0.24 | [-0.12, 0.84] | .14 |
| Others × 2021 | 0.11 | 0.32 | [-0.53, 0.75] | .74 | 0.25 | 0.36 | [-0.46, 0.97] | .49 |
| Others × 2022 | -0.43 | 0.33 | [-1.07, 0.21] | .19 | -0.47 | 0.36 | [-1.18, 0.24] | .20 |
| **Age at breast cancer diagnosis,**  **per 5-year increase** |  |  |  |  | -0.058 | 0.040 | [-0.14, 0.020] | .14 |
| **Years from diagnosis to survey,**  **per 1-year increase** |  |  |  |  | -0.0072 | 0.01 | [-0.036, 0.021] | .62 |
| **No. of people living together,**  **per 1-person increase** |  |  |  |  | -0.12 | 0.05 | [-0.23, -0.020] | .020 |
| **Marital status (ref: married/living with partner)** |  |  |  |  |  |  |  |  |
| Single/never married |  |  |  |  | 0.95 | 0.25 | [0.46, 1.44] | < .001 |
| Divorced, separated, or widowed |  |  |  |  | 0.40 | 0.18 | [0.041, 0.76] | .029 |
| **Insurance type (ref: private insurance)** |  |  |  |  |  |  |  |  |
| Medicare |  |  |  |  | 0.21 | 0.22 | [-0.22, 0.64] | .35 |
| Medicaid |  |  |  |  | 0.93 | 0.35 | [0.23, 1.62] | .009 |
| Others |  |  |  |  | -0.27 | 0.45 | [-1.15, 0.60] | .54 |
| Abbreviations: S.E., standard error; CI, confidence interval; ref, reference; BCS, breast cancer survivors; GED, General Educational Diploma.  Models 4 and 5 were set up similarly as Models 1 and 2 in Table 2, with the outcome variable changed to the social isolation score. As described in Methods, this score was based on responses to the first four question items: “How isolated are you feeling now due to COVID-19?”, “Do you feel supported by friends and family during this time of COVID-19?”, “How optimistic are you that you are supported to manage life’s challenges successfully?” and “If you need medical help, how confident are you that you can find someone to take you to the doctor's office?” The socioeconomic covariates were selected if they had a statistically significant association with the new outcome in univariate analyses, so the confounders being adjusted in the final multivariate model were not exactly the same as in Table 2. | | | | | | | | |

**Supplemental Table 6.** Mixed-effects Models of the **Stress Score** with Interactions between Survey Year and Race/Ethnicity

|  | **Model 6: Racial Disparities over Time**  **in the Stress Score** | | | | **Model 7: Racial Disparities over Time**  **in the Stress Score**  **Further Adjusting for Potential Confounders** | | | |
| --- | --- | --- | --- | --- | --- | --- | --- | --- |
|  | **Coefficient** | **S.E.** | **95% CI** | ***P*** | **Coefficient** | **S.E.** | **95% CI** | ***P*** |
| **Racial/ethnic group (ref: White BCS)** |  |  |  |  |  |  |  |  |
| Black BCS | -0.20 | 0.28 | [-0.75, 0.35] | .48 | -0.31 | 0.32 | [-0.94, 0.33] | .34 |
| Others | 1.26 | 0.47 | [0.35, 2.18] | .007 | 0.72 | 0.49 | [-0.24, 1.67] | .14 |
| **Survey year (ref: 2020)** |  |  |  |  |  |  |  |  |
| 2021 | -0.69 | 0.13 | [-0.95, -0.44] | < .001 | -0.66 | 0.13 | [-0.93, -0.40] | < .001 |
| 2022 | -1.20 | 0.13 | [-1.45, -0.94] | < .001 | -1.14 | 0.14 | [-1.41, -0.87] | < .001 |
| **Racial/ethnic group × Survey year interaction**  **(ref: White BCS × 2020)** |  |  |  |  |  |  |  |  |
| Black BCS × 2021 | 0.89 | 0.27 | [0.35, 1.42] | .001 | 0.67 | 0.29 | [0.10, 1.24] | .021 |
| Black BCS × 2022 | 0.90 | 0.28 | [0.34, 1.45] | .002 | 0.73 | 0.30 | [0.14, 1.32] | .015 |
| Others × 2021 | 0.14 | 0.43 | [-0.70, 0.99] | .74 | 0.25 | 0.44 | [-0.62, 1.12] | .57 |
| Others × 2022 | -0.26 | 0.43 | [-1.11, 0.58] | .54 | -0.23 | 0.45 | [-1.11, 0.64] | .60 |
| **Age at breast cancer diagnosis,**  **per 5-year increase** |  |  |  |  | -0.40 | 0.058 | [-0.52, -0.29] | < .001 |
| **Years from diagnosis to survey,**  **per 1-year increase** |  |  |  |  | -0.027 | 0.02 | [-0.069, 0.014] | .20 |
| **Insurance type (ref: private insurance)** |  |  |  |  |  |  |  |  |
| Medicare |  |  |  |  | 0.68 | 0.34 | [0.010, 1.34] | .047 |
| Medicaid |  |  |  |  | 1.78 | 0.50 | [0.80, 2.76] | < .001 |
| Others |  |  |  |  | 0.16 | 0.69 | [-1.19, 1.50] | .82 |
| **Education level (ref: graduate/professional degree)** |  |  |  |  |  |  |  |  |
| Bachelor’s degree |  |  |  |  | 0.15 | 0.25 | [-0.35, 0.65] | .56 |
| Trade/technical school, some college, Associate’s |  |  |  |  | -0.16 | 0.27 | [-0.70, 0.37] | .55 |
| High school graduate/GED |  |  |  |  | -0.49 | 0.37 | [-1.21, 0.24] | .19 |
| Did not finish high school |  |  |  |  | 3.70 | 1.05 | [1.64, 5.75] | < .001 |
| Abbreviations: S.E., standard error; CI, confidence interval; ref, reference; BCS, breast cancer survivors; GED, General Educational Diploma.  Models 6 and 7 were set up similarly as Models 1 and 2 in Table 2, with the outcome variable changed to the stress score. As described in Methods, this score was based on responses to the last seven question items: "Did you feel you could keep up with your work and home responsibilities (including working from home now)?", "Have you felt fearful?", "Have your worries overwhelmed you?", "How much did you worry about getting sick from COVID-19?", "How often were you worried that you might need to go to the hospital?", "Lately, have you felt hopeful?" and “In the past month, to what extent have you felt nervous or stressed?" The socioeconomic covariates were selected if they had a statistically significant association with the new outcome in univariate analyses, so the confounders being adjusted in the final multivariate model were not exactly the same as in Table 2. | | | | | | | | |
